# How to: Success factors for the implementation and establishment of the “longitudinal curriculum” on communicative competencies at the Medical Faculty Mannheim 

**DOI:** 10.3205/zma001593

**Published:** 2023-02-15

**Authors:** Renate Strohmer, Ute Linder, Jens J. Kaden

**Affiliations:** 1 Medizinische Fakultät Mannheim, Universität Heidelberg, Geschäftsbereich Studium und Lehrentwicklung, Lernkrankenhaus TheSiMa, Mannheim, Germany

**Keywords:** longitudinal communication curriculum, curriculum development, communicative competencies, how to

## Abstract

Communicative competencies are of great importance to the medical profession, hence the teaching of them has been continuously expanded in recent years at many German medical schools. While individual courses on communicative competencies have already been established in the curricula, there remains, in part, a lack of longitudinal anchoring over the entire course of medical study.

In 2008 the Medical Faculty Mannheim began implementing a longitudinal curriculum for communicative competencies. This paper outlines the general and phase-specific success factors in this process and gives practical recommendations and tips based on the personal experiences of the authors and the existing literature.

## Abstract (de)

Kommunikative Fähigkeiten sind für den Arztberuf von großer Bedeutung und dementsprechend wird die Lehre hierzu insbesondere in den letzten Jahren an vielen Medizinischen Fakultäten fortwährend ausgebaut. Während einzelne Lehrveranstaltungen zur Vermittlung kommunikativer Kompetenzen in den Curricula bereits etabliert sind, mangelt es teilweise noch an einer longitudinalen Verankerung über das gesamte Studium der Humanmedizin hinweg.

An der Medizinischen Fakultät Mannheim wurde bereits 2008 mit der Implementierung eines longitudinalen Curriculums „kommunikative Kompetenzen“ begonnen. In diesem Artikel werden übergreifende und phasenbezogene Erfolgsfaktoren dieses Prozesses nachgezeichnet und praxisbezogene Handlungsvorschläge und Tipps – basierend auf den persönlichen Erfahrungen der Autor*innen sowie der verfügbaren Literatur – gegeben.

## 1. Background

Communicative competence is defined as “the ability to achieve communicative goals in a socially appropriate manner” [1]. This can be taught and learned through practice and experience (ibid.). A physician’s ability to communicate can contribute significantly to a patient’s recovery [2], [3], [4], [5], e. g., to reduce symptoms and increase quality of life [6].

Teaching and mastering communicative competence are considered an important pillar of medical education and identified in the medical licensing regulations [http://www.gesetze-im-internet.de/_appro_2002/index.html]. In the draft legislation drawn up by the Federal Ministry of Health to revise medical education, holding a medical consultation with a patient is defined as a main area of competence [7]. Learning objectives for holding medical consultations are defined in the National Competency-based Catalogue of Learning Objectives for Undergraduate Medical Education (NKLM) [https://www.nklm.de]. One goal of the 2020 Masterplan for Medical Studies is the implementation of a national longitudinal communication curriculum in medicine [8]. Reports from different medical schools in Germany were published, among other papers, in the GMS JME special topic issue on “communicative and social competencies” [9] and success factors have been identified [10], which can also be found, in part, in the Undeloher recommendations for curriculum development [11].

This paper picks up on the previous papers because the implementation of the curriculum in Mannheim is now quite advanced and a range of experiences has been gathered, primarily with regular operation and the support and maintenance of the teaching program, and from which concrete recommendations can be drawn.

The Mannheimer reformed medical curriculum (MaReCuM), a model study program in medicine, was introduced at the Medical Faculty Mannheim of the University of Heidelberg in 2006 and augmented in 2008 with a longitudinal curriculum in communicative competencies. Communicating with patients, their families, and other colleagues forms one of the seven core competencies that are taught by MaReCuM. From the first semester onward, communication is taught, learned and tested. During the first study phase (years 1-2), basic theoretical knowledge, tools and approaches are taught and practiced in small student groups. During the second study phase (years 3-5), there are course units with simulated persons (SP), to which the previously learned tools and techniques can be applied. These conversations are successively built upon in terms of their content and complexity and culminate in communication with real patients during bedside teaching and the final practical phase of medical study (year 6).

The building blocks for successful curriculum implementation and the central factors for success – identified retrospectively at the Medical Faculty Mannheim – are presented in the following. These are addressed in chronological order from conception and implementation through to regular operation. Factors that pertain to more than one phase are also identified and compared with the existing literature.

## 2. Success factors for a longitudinal communication curriculum

### 2.1. Success factors applicable to multiple phases

#### 2.1.1. Centralized coordination

First of all, a central point for coordination was defined at the Medical Faculty Mannheim to ensure the complex process of creating and sustaining a longitudinal curriculum on communicative competency. This central point represents the contact partner for all participants, organizes the design-related tasks, works, in part, on the conception and design, bundles the quality management (particularly during regular operation) and enables cross-linkage–e. g., to scientific committees. In Mannheim this central point is housed at the academic teaching hospital, TheSiMa (Themenräume-Simulation-Mannheim). An additional staff member (50% full-time equivalent), funded with project money, was hired for a period of two years to exclusively handle the conceptual work on the communicative curriculum.

The acceptance of this central point is an especially critical success factor in terms of the process, since it ensures that all of the information comes together in one place and that specific work steps can be centrally managed. Failing this, it is easy for individual course units to be offered in different semesters that may be qualitatively high in value, but are not coordinated with each other longitudinally.

##### 2.1.2. Organizational support and identification

A longitudinal curriculum affects many areas of teaching and requires networking and cooperation among many participants. This necessitates coordination and compromises. Ensuring the commitment of all of the participants not only to the process, but also to the significance of teaching communication itself, is therefore another key factor for success [11]. This requires that all of the participants and the decision-makers responsible for the process are committed to the project, recognize its importance, want to and can carry responsibility for it. Other authors also refer to this as the vision or the urgency that must be seen or felt by all of the participants [12]. In addition to those involved with the internal process, it is important to secure the support of other officials and stakeholders, for instance, university deans, study committees, and student bodies.

While developing the longitudinal communication curriculum in Mannheim, a basic tenor was present, due among other things to the prior development of the reformed curriculum, regarding the value of such a project and this was clearly conveyed by those in leadership positions. Also, early and active inclusion of the people responsible for the modules served to strengthen the level of commitment. Stakeholders were regularly informed through a variety of channels, per email and in personal conversations and meetings. The central stakeholders were reached in this manner and share responsibility for the curriculum through to this day; they support its continued development and are important links to their institutes, hospitals and other institutions.

#### 2.2. The planning and implementation phase

##### 2.2.1. Working group

The basis for the content development of a longitudinal curriculum is formed by the cooperation between many different academic departments, institutes and hospitals. To enable this, the formation of a steering committee [11] or a working group [12] in terms of an academic advisory committee is recommended. Interdisciplinary and interprofessional collaboration is important not only in regard to the actual development of curricular content, but also as a pre-requisite for the success of the implementation process itself. Furthermore, such a group, composed of established and permanently responsible persons, can strengthen the approval for the project.

At the Medical Faculty Mannheim, the interprofessional working group is composed of persons responsible for the modules and teaching, those in leadership positions at hospitals, faculty heads and employees of the teaching hospital and SP program. The working group receives essential support from the Office of the Dean of Studies. There was no compensation for the work. The members of the working group joined the project out of an intrinsic motivation, for example, due to an interest in the topic or a conviction about its importance.

At the start of the planning phase, the working group had the support of an external expert on communication training. Following a kick-off meeting, two workshops were held on different topics, such as methods training, communication models, group dynamics, and conversational tools. These workshops were meant to inspire content and expand the knowledge of the participants – they also served on a meta-level as a team-building measure for the working group. After the workshops, there were other meetings to establish consensus regarding the curriculum.

##### 2.2.2. Developing the curriculum (actual-target analysis)

Generally speaking, a large number of courses already have content for teaching communication to medical students. It is important to take an inventory so as to make this visible and to recognize redundancies and missing content. Curricular mapping according to Harden [10], [13] or a survey instrument for structure analysis [14] can be used to accomplish this. At the Medical Faculty Mannheim, the actual situation was mapped through reverse blueprinting by the project staff member at the centralized coordination point. The existing teaching and learning materials were reviewed for content and documented; course observations were carried out, and module coordinators and other people responsible at the subject level were interviewed. This resulted in a blueprint showing learning objectives, competencies, content and methods. This mapping was done over a period of about a year. Cooperation took place smoothly because the process was initiated and communicated from a central point.

As soon as the actual situation has been assessed, it is important to compare it with a defined target situation [12]. The learning objectives in the NKLM or the sample curriculum for communication in medicine [15] can be used as targets. By doing this, it is possible to determine if all of the individual learning objectives relevant to physician communication are covered or lacking in content or present in multiple forms. These results can be compiled and documented in a module manual and differentiated up through each individual course session. This makes it clear which learning objective should be taught using which content or tested at which time point and in which form.

A content-based comparison between the actual curricular content and methods and the targets was conducted at the Medical Faculty Mannheim based on the inventory analysis done in the conception phase. To do this, the Canadian CanMEDS framework [16] and the Skills Overview of the GMA committee on practical skills [17] were used, among other things, and the expertise of the working group members was drawn upon. This approach was selected because, at that point in time, the NKLM had not yet been adopted and the IMPP had not yet completed its nationwide Longkomm project. Finally, to develop a logical and coherent curricular thread, working group meetings were held, during which the results of the mapping exercise were discussed and individual courses were defined in terms of their type, scope and specific content.

Focusing the curriculum on specific content elements has proven effective, for instance, the implementation of specific feedback rules [18] and tools such as RE-Form (guidelines on opening conversations) [19], NURSE (dealing with emotions) [20], [21], [22] and SPIKES (delivering bad news) [23], [24]. This content is anchored longitudinally in terms of chronology and cross-sectionally according to content and subjects (see table 1 (Tab. 1)). Furthermore, the Medical Faculty Mannheim has placed and still places value on the fact that clinically active physicians from all disciplines teach communication courses as a way to emphasize the importance of physician communication in all clinical subjects.

During the first two years of study basic theoretical knowledge is taught in lectures and seminars on the role of the physician, physician communication and building a relationship. This is repeated and covered in more depth in seminars. In the third year of study there are seminars on migration/health, psychosocial background, the doctor/patient relationship, holding medical consultations with patients, and handling emotions. These acquired techniques are practiced and honed in a simulated consultation with simulated patients. In the fourth year of study there are seminars on delivering bad news, behavior modification, and suicidality; these are also practiced with SPs. After this come the first consultations with real patients. During the fifth year of study there are simulated consultations in pediatrics on the topic of conflict management and in primary care on the language barriers faced in consultations with patients who have migrant backgrounds.

##### 2.2.3. Designing assessments

As part of constructive alignment [25], teaching and learning methods, learning objectives and the related test formats should be constructed so that they reflect each other. This means that practically acquired competencies should also be tested by means of a practical assessment. For this reason, two summative Objective Structured Clinical Examinations (OSCE) were implemented in MaReCuM to assess communicative competencies.

The topic of communication is integrated into the third-year OSCE. In addition to a station dedicated solely to communication, two of 12 stations include communicative tasks as part of the assignments.

The OSCE that assesses interdisciplinary learning in the fifth year of study includes communicative competence in the evaluation: Abilities to communicate in medical consultations account for 25% of an examinee's score, whereby a communication-relevant task is integrated into each of the 12 stations. The high value placed on physician communication is reinforced, as a result and also in respect to the final practical year of medical study.

The structures and courses in the longitudinal communication curriculum at the Medical Faculty Mannheim were compiled as a manual in an easy-to-handle booklet form. This contains information on the tools and strategies taught for holding medical consultations and additional sources on this topic. The manual is issued to students and teachers and updated on a regular basis.

##### 2.2.4. Structuring the teaching

For (external) teachers who are involved mainly in their capacity as physicians, the realization of teaching students as part of a weekly schedule in the context of tightly scheduled hospital routines is often difficult.

To make teaching communication more attractive to these physician teachers, planning block courses and day-long teaching blocks, for instance, has proven more attractive in Mannheim than weekly course sessions. This also makes it possible to recruit external instructors for this type of teaching since they find the commute very worthwhile for an entire day of teaching.

##### 2.2.5. Teacher training

Teachers often bring with them a variety of assumptions about pedagogy, content and motivation to the structure and design of courses on medical consultation skills, a situation that make teacher qualification necessary for this type of teaching format [x]. To ensure a consistently high and homogenous quality of instruction, the Medical Faculty Mannheim designed and launched required, multi-step, modular teacher trainings early on as preparation for teaching with SP. At first, this involved in-person training sessions. As of 2016, in response to the participants' complaints about scheduling, a more flexible blended-learning program has been offered and covers small learning units in bite-size format [26], [27]. Thus, the teacher training has been adapted to fit the needs of the teachers – particularly in terms of time and scheduling – and is highly accepted. As part of the teacher training, the participants first go through online training that concludes with a test of knowledge. Once this test has been passed, the participants are cleared to attend the in-person training. Teacher training ends with a formal observation of them while teaching with feedback afterward.

In addition to basic qualification, teachers can seek qualification in more specialized subjects in the communication curriculum (behavior modification, delivering bad news, etc.). All of the training culminates in a certificate, which is required to teach communication courses (with SP). The bite-sized structure of the training program coupled with the online format also gives teachers the option to repeat any of the modules, as needed.

Specific training is also required for the examiners who serve as assessors in the OSCEs referred to above [28]. By putting emphasis on training that takes the needs of the instructors into account, it is not only possible to improve the quality of the teaching, but also boost the approval of the teachers.

Other authors have cited the importance of teacher training and manuals for standardization and quality assurance in the teaching of communication courses [12], and this has been broadened to include factors such as ongoing training and the expansion of teaching-learning research [10].

#### 2.3. Regular operation

##### 2.3.1. Maintaining operation of the teaching program

Realizing the teaching of courses on medical consultation skills represents a central task of normal operation. Belonging to this are creating the course schedules and preparing the individual seminars. This also encompasses the assignment of teachers and SPs to specific courses. Course materials, online content and training sessions must be kept up to date and expanded or revised. At the Medical Faculty Mannheim, teachers are given access to course files containing the course syllabus in table form, the required teaching materials and assignments, and observation criteria for the simulated consultations. These files serve to homogenize the instruction and assure quality. These files are provided by the central coordination point for all of the courses offered at TheSiMa. The basic studies program is administered by another office.

##### 2.3.2. Recruiting teachers

The acquisition of new teachers is another task associated with regular operation. Teacher fluctuation must be monitored and counteracted through the targeted recruitment of new teachers. In Mannheim, extensive communication between the central point and hospitals, those responsible for the modules, and the Office of the Dean of Studies has proven constructive. What has shown itself to be particularly helpful is contacting these partners directly and forwarding a request for new teachers to the physicians who have already been given a teaching assignment.

##### 2.3.3. Quality assurance

Under regular operation, assuring the quality of courses in communicative competencies forms a core task. At the Medical Faculty Mannheim this is ensured through many activities. First, the courses are regularly evaluated within the scope of the school-wide and university-wide evaluations using standardized questionnaires. In addition, the suggestions and feedback elicited from the teachers and students regarding the courses are used to adapt course content and teaching methods. Course materials are regularly updated by the central coordination point. Also, random teacher observations are carried out intermittently, in which the teaching is compared to the prescribed setting. The teachers receive feedback directly afterward. Feedback from the SPs regarding the quality of the instruction is also responded to.

The longitudinal communication curriculum itself is updated and adapted in regular reviews held by the central coordination, the members of the working group and their successors.

## 3. Conclusion

The experience of developing and implementing a longitudinal curriculum on communicative competencies at the Medical Faculty Mannheim shows that this process requires effort, resources and can be protracted. Maintaining regular teaching operation after the implementation phase and its associated tasks should not be underestimated. Taking success factors into account during the different phases of implementing a communication curriculum can, in our opinion, contribute to the success of the process and to the development of quality in medical education. In our view, these factors entail:

- establishing a centralized coordination point,
- ensuring organizational support,
- forming a working group,
- structured development of a curriculum,
- structuring the teaching,
- recruiting and training the teachers,
- maintaining the teaching program,
- established quality assurance.

## Competing interests

The authors declare that they have no competing interests. 

## Figures and Tables

**Table 1:**
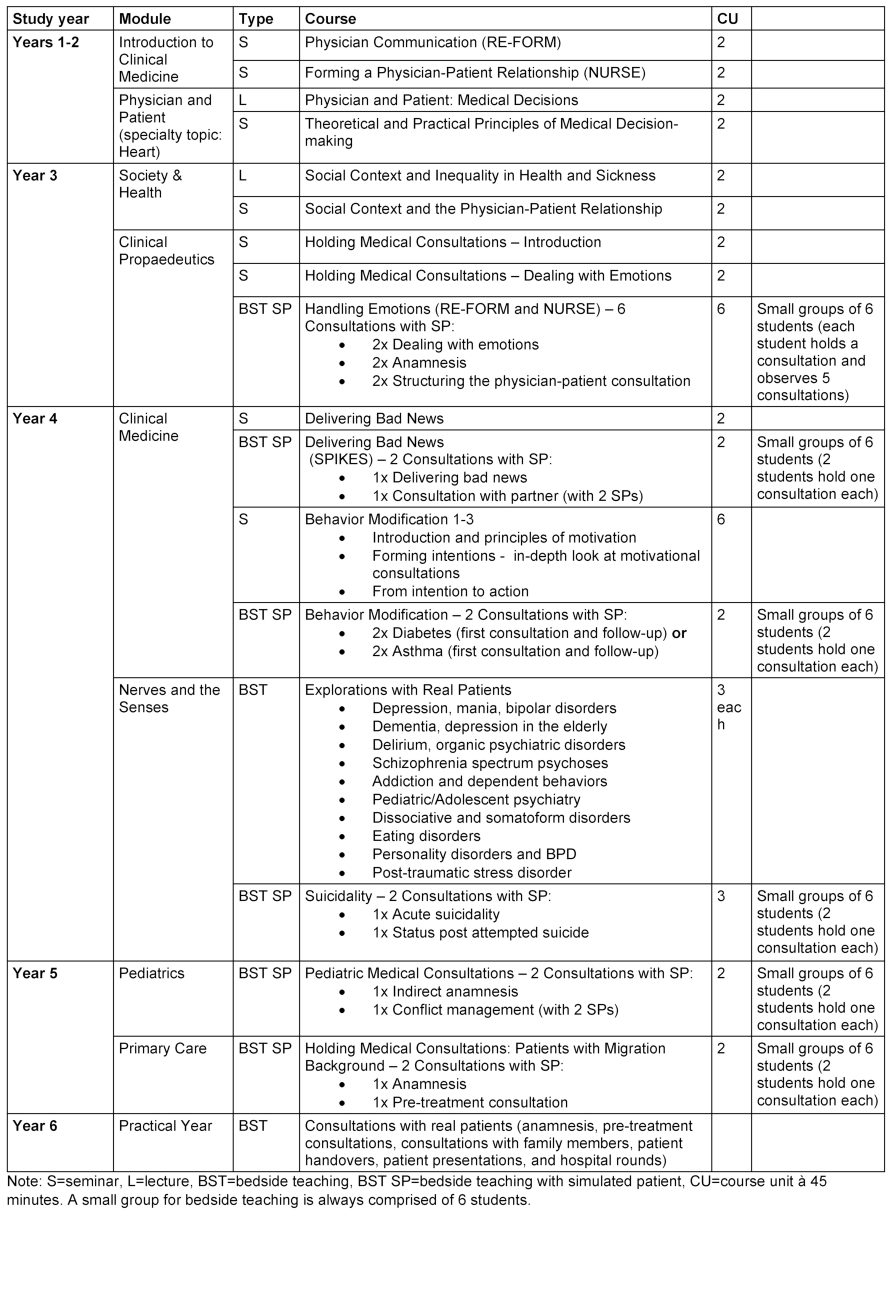
Courses in the longitudinal curriculum on communicative competencies in MaReCuM
